# Invasive *Salmonella* infections among children in Bintulu, Sarawak, Malaysian Borneo: a 6-year retrospective review

**DOI:** 10.1186/s12879-019-3963-x

**Published:** 2019-04-18

**Authors:** Anand Mohan, Chandran Munusamy, Yee-Chin Tan, Sobana Muthuvelu, Rohaidah Hashim, Su-Lin Chien, Ming-Kui Wong, Nurul Aiman Khairuddin, Yuwana Podin, Peter Sie-Teck Lau, David Chun-Ern Ng, Mong-How Ooi

**Affiliations:** 1 Department of Paediatrics, Bintulu Hospital, Bintulu, Sarawak Malaysia; 2 Institute of Health and Community Medicine, Universiti Malaysia Sarawak, Kota Samarahan, Sarawak Malaysia; 3 Bacteriology Unit, Infectious Disease Research Centre, Institute for Medical Research, Kuala Lumpur, Malaysia; 4 Department of Pathology, Bintulu Hospital, Bintulu, Sarawak Malaysia; 5 Department of Paediatrics, Hospital Tuanku Ja’afar, Seremban, Negeri Sembilan Malaysia; 6 Department of Paediatrics, Sarawak General Hospital, Kuching, Sarawak Malaysia

**Keywords:** *Salmonella*, Invasive, Non-typhoidal, Children, Malaysia, Borneo

## Abstract

**Background:**

Invasive *Salmonella* infections result in significant morbidity and mortality in developing countries. In Asia, typhoid and paratyphoid fever are reported to be the major invasive *Salmonella* infections, while invasive non-typhoidal *Salmonella* (iNTS) infections are believed to be uncommon. Data from Sarawak, in Malaysian Borneo, are limited.

**Methods:**

A retrospective study identifying all children aged < 15 years with invasive *Salmonella* infections from 2011 to 2016 was conducted in Bintulu Hospital in Sarawak. Population incidences, clinical and bacterial characteristics were examined.

**Results:**

Forty-four patients were identified during the 6-year study period: 43 had iNTS infection and 1 had typhoid fever. The average annual iNTS incidence was 32.4 per 100,000 children aged < 5 years. None of the children had malaria or HIV infection, and only 7% were severely malnourished. *Salmonella* Enteritidis and *Salmonella* Java were the commonest NTS serovars identified. Pneumonia was the most common manifestation of iNTS disease, present in 20 (47%) children. Other manifestations included gastroenteritis, fever without a source, septic arthritis and meningitis. *Salmonella* Enteritidis was identified in 76% of those with pneumonia, significantly more frequently than in children with other manifestations. Over 25% of children with iNTS developed severe disease and nearly 10% suffered long term morbidity or mortality. While 78% of *Salmonella* Java isolates were multi-drug resistant, nearly all other isolates were susceptible to most antimicrobials, including ampicillin.

**Conclusions:**

Bintulu Division in Sarawak observed a very high incidence of childhood iNTS infections. Enteric fever was uncommon. The epidemiology of invasive *Salmonella* infections in Malaysian Borneo differs considerably from that of neighbouring countries in Asia.

## Background

Invasive *Salmonella* infections, caused by the various serovars of *Salmonella enterica* subspecies enterica, result in significant morbidity and mortality in developing countries [1, 2]. *Salmonella enterica* serovars Typhi and Paratyphi, known as typhoidal *Salmonella*, typically cause the well-known manifestations of enteric fever (typhoid or paratyphoid fever) [1]. Other *Salmonella enterica* serovars, collectively termed non-typhoidal *Salmonella* (NTS), are important causes of gastro-intestinal infections, but may also cause severe invasive infections including bacteraemia and meningitis, with high fatality rates [3, 4]. These invasive NTS (iNTS) infections have mainly been reported in sub-Saharan Africa, predominantly among children with malnutrition or malaria and in adults living with human immunodeficiency virus (HIV) infection [5]. NTS have now emerged as a leading cause of bacterial bloodstream infection among children with fever in the sub-Saharan region, with population incidences as high as 742 per 100,000 person-years of observation [6].

The increasing iNTS burden seen in African children has not been mirrored in Asia. A large multicentre prospective study involving 5 Asian countries reported only 5 paediatric iNTS cases in its 12 month surveillance period [7]. The same study confirmed that typhoid and paratyphoid fever remain the predominant invasive *Salmonella* infections in Asian children, with annual incidences in Indonesia, India and Pakistan ranging from 149 to 573 per 100,000 children aged 2–5 years [8]. Recently however, a study in Kuala Lumpur in peninsular Malaysia reported that 16% of childhood community-acquired bacteraemia cases were caused by NTS, whereas only 2% were due to typhoidal *Salmonella* [9]. Unfortunately, unlike in Africa, little is known regarding the characteristics of these iNTS infections.

The burden of both typhoidal and iNTS infections among children in Sarawak, Malaysian Borneo is not known. Similarly, the prevailing NTS serovars, risk factors for infection, clinical manifestations and outcomes, and the anti-microbial susceptibility of serovars in the region have never been documented. To answer these questions, we conducted a retrospective chart review to determine the population incidence, the clinical and the bacterial characteristics of invasive *Salmonella* infections among children in Bintulu, Sarawak.

## Methods

### Study site

The study was conducted at Bintulu Hospital, a 294 bed district hospital which provides medical, surgical and intensive care services to adults and children living in Bintulu Division and Belaga district of Kapit Division in Sarawak, Malaysian Borneo. This 32,000km^2^ area located within the central region of the state has a total population of approximately 256,000, including 72,000 children < 15 years. A large proportion of this population resides in Bintulu town, the largest town in the region. The region has a tropical climate with monsoon rains occurring between November and February each year. Malaysia has low HIV infection and malaria disease burdens, with incidence rates of 10.7 and 7.3 per 100,000 population reported in 2016 respectively [10].

### Case definitions

An invasive *Salmonella* infection case was defined as a child < 15 years with a positive culture of *Salmonella enterica* species from any normally sterile site sample obtained within 48 h of admission. We retrospectively identified all cases admitted from January 2011 to December 2016 through a manual search of the microbiology laboratory logbooks and electronic database. Medical records of identified cases were then retrieved and details on demography, underlying medical conditions, symptoms, physical findings, laboratory results, case management, outcome and bacterial antimicrobial susceptibility and serotype were collected using standardized case report forms.

NTS included all *Salmonella enterica* subspecies enterica serotypes other than Typhi and Paratyphi A. In children aged < 10 years, severe malnutrition was defined as weight for age z-score ≤ − 3 based on the World Health Organization (WHO) child growth standards and the WHO growth reference data [11, 12]. Children aged > 10 years were excluded from this analysis. A clinical diagnosis of pneumonia was made when fever, cough and tachypnoea were present. Meningitis was confirmed when culture of cerebrospinal fluid (CSF) yielded *S. enterica* or when CSF examination revealed pleocytosis (≥10 white blood cells/μL in a non-traumatic lumbar puncture) in the presence of *Salmonella* bacteraemia. Tachycardia and tachypnoea were defined based on WHO definitions [13]. Severe anaemia was defined as a haemoglobin level < 7 g/dL. Multi-drug resistance was defined as non-susceptibility to 3 or more unrelated antimicrobial agents. Intermediate resistant isolates were considered non-susceptible. An appropriate antibiotic was defined as the use of an antimicrobial with good intracellular activity, namely amoxicillin, ampicillin, cotrimoxazole, chloramphenicol, ciprofloxacin or a 3rd generation cephalosporin, to which the *Salmonella* isolate was susceptible. A recurrent infection was defined as a culture-confirmed *Salmonella* infection occurring after the completion of an appropriate antibiotic for a previous invasive *Salmonella* infection.

### Microbiological methods

As part of routine clinical practice in Bintulu Hospital, blood for bacterial culture was collected in all children suspected to have severe bacterial infections and in those requiring parenteral antibiotics, based on the discretion of admitting clinicians. Between 1 and 3 ml of whole blood was collected and immediately inoculated into paediatric bottles (BACTEC Peds Plus/F Medium; Becton Dickinson, USA) and incubated in an automated blood culture system (BACTEC FX; Becton Dickinson, USA) for a maximum of 5 days. Positive growth was subcultured onto blood agar, chocolate agar and MacConkey agar. CSF and endotracheal secretions were cultured directly on blood agar, chocolate agar and MacConkey agar, while urine samples were cultured on blood agar and cystine lactose electrolyte deficient agar. *Salmonella enterica* was identified with either API®20E (BioMérieux, France) or BBL™ Crystal™ Identification Systems (Becton Dickinson, USA).

Antibiotic susceptibility were determined by Kirby-Bauer disk diffusion test (Becton Dickinson, USA) according to the Clinical and Laboratory Standard Institute (CLSI) guidelines. Breakpoints were based on the 2008 guidelines for isolates obtained prior to September 2013, and on the 2011 guidelines for isolates obtained subsequently.

Serotyping of the *Salmonella* isolates were performed in the national reference laboratory (Institute for Medical Research, Kuala Lumpur) according to the Kauffman-White scheme. Slide agglutination tests using commercially available mono- and poly-O groups *Salmonella* A, B, C, D and E antisera (Remel, Europe Ltd., UK) and polyvalent *Salmonella* antisera phase 1 and phase 2 flagellar H antigens (Remel, Europe Ltd., UK) were performed as described previously [14]. *Salmonella enterica* serovar Paratyphi B var. Java was distinguished from classical *Salmonella* Paratyphi B using a multiplex PCR targeting the common region for *Salmonella* and the ATG start codon for the STM 3356 gene of d-tartrate fermenting strains, as described previously [15].

HIV and malaria screening were only performed if clinically suspected.

### Statistical analysis

Statistical analysis was performed using SPSS Statistics 21. The Kruskal-Wallis *H* test was used for numerical variables and either the χ^2^ test or Fisher’s exact test was used for categorical variables. The correlation between the number of cases and average monthly rainfall was examined using Spearman’s rank co-efficient test. Population data were obtained from the Malaysian Census Data 2010. Meteorological data were obtained from the Malaysian Meteorological Department.

### Ethics statement

The study was approved by the Malaysian Medical Research Ethics Committee (NMRR-17-2438-37563). All data analyzed were anonymized.

## Results

### Bacterial isolation and typing

Forty-four children with invasive *Salmonella* infections were identified during the 6-year study period (3 in 2011, 4 in 2012, 9 in 2013, 7 in 2014, 13 in 2015 and 8 in 2016). Medical records of all identified cases were available for analysis. All cases had *Salmonella* isolated from blood. *Salmonella* was not isolated in any other specimen, including CSF.

**Fig. 1:**
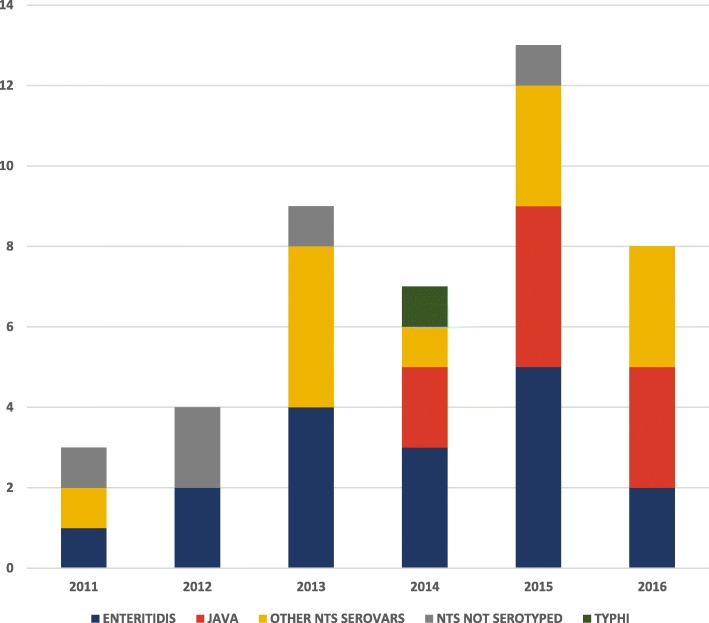
Distribution of the 44 invasive *Salmonella* cases by year of admission and serovar. The bar chart shows the number of invasive *Salmonella* cases diagnosed in Bintulu in each year from 2011 to 2016, according to serovar

NTS was isolated in 43 (98%) children while *Salmonella enterica* serovar Typhi was isolated in only 1 (2%) child. Of the 38 (88%) NTS isolates that were serotyped, *Salmonella enterica* serovar Enteritidis and *Salmonella enterica* serovar Paratyphi B var. Java (*Salmonella* Java) were the most common serovars identified (Table 1). *Salmonella* Java infections were only noted from 2014 (Fig. 1).

**Table 1:** Distribution of non-typhoidal *Salmonella* serovars in Bintulu

Serovar	Number	Percent
*Salmonella* Enteritidis	17	45
*Salmonella* Java	9	24
*Salmonella* Corvallis	6	16
*Salmonella* Ohio	1	3
*Salmonella* Kentucky	1	3
*Salmonella* Poona	1	3
*Salmonella* Derby	1	3
*Salmonella* Agona	1	3
*Salmonella* Hvittingfoss	1	3
Total	38	100

### Patient demographics and population incidence

Twenty-eight (64%) of the 44 children with invasive *Salmonella* infections were male. The median age of children with iNTS was 8 months (interquartile range [IQR], 4–11 months; range 8 days-11.8 years). Seven (16%) were aged < 2 months. The child with typhoid was 54 months old.

The average annual incidence of iNTS was 10.0 per 100,000 children aged < 15 years, with the highest incidence recorded in 2015 (18.1 per 100,000 children per year). The average annual incidence was 32.4 per 100,000 in children aged < 5 years and 135.1 per 100,000 in those aged < 1 year. In contrast, the average annual incidence of *Salmonella* Typhi infection was only 0.2 per 100,000 children < 15 years.

**Fig. 2:**
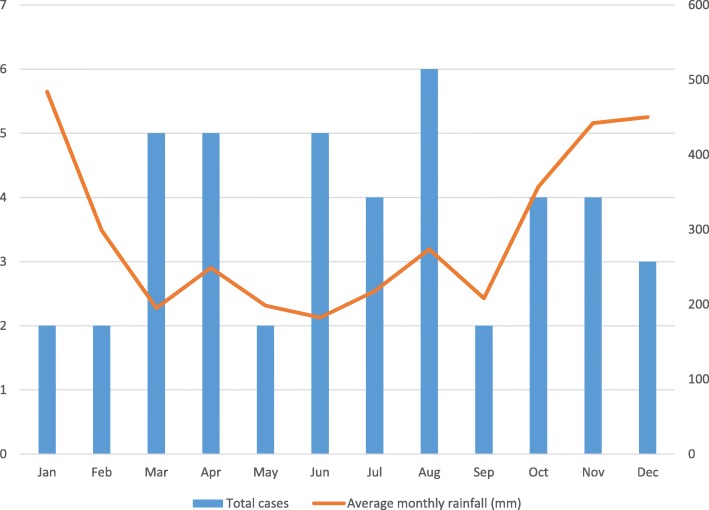
Distribution of the 44 invasive *Salmonella* cases and average rainfall by month. The bar chart shows the distribution of the 44 invasive *Salmonella* cases according to the month of admission. The average monthly rainfall over the 6-year period (January 2011–December 2016) in Bintulu is shown in the line graph

Although more invasive *Salmonella* cases occurred during the dryer months of the year (Fig. 2), no significant correlation was found between the monthly incidence and average monthly rainfall (*r*_*s*_ = − 0.3, *P* = 0.3).

### Risk factors and clinical manifestations

Thirteen (30%) children had underlying medical conditions (Table 2), including 1 child who was diagnosed to have acute leukemia at presentation. None were suspected or diagnosed to have malaria or HIV infection. Severe malnutrition was present in only 3 (7%) of the 43 children aged < 10 years. Of these, 2 had *Salmonella* Enteritidis and 1 had *Salmonella* Java infection. Severe anemia was present in only 2 (5%) of the 43 children with data available. Both had *Salmonella* Enteritidis infection.

**Table 2:** Underlying medical conditions among children with invasive *Salmonella* infections in Bintulu

Medical condition	Number	% of all cases	NTS serovar isolated (number of cases)
Episodic viral wheeze/ multi-trigger wheeze	5	11	*Salmonella* Enteritidis (2), *Salmonella* Corvallis (1), Not serotyped (2)
Down syndrome	2	5	*Salmonella* Enteritidis (2)
Acute leukaemia	2	5	*Salmonella* Enteritidis (2)
Eczema	2	5	*Salmonella* Java (1), *Salmonella* Corvallis (1)
West syndrome	1	2	*Salmonella* Ohio (1)
Neonatal hepatitis syndrome	1	2	*Salmonella* Enteritidis (1)
Total	13	30	

**Fig. 3:**
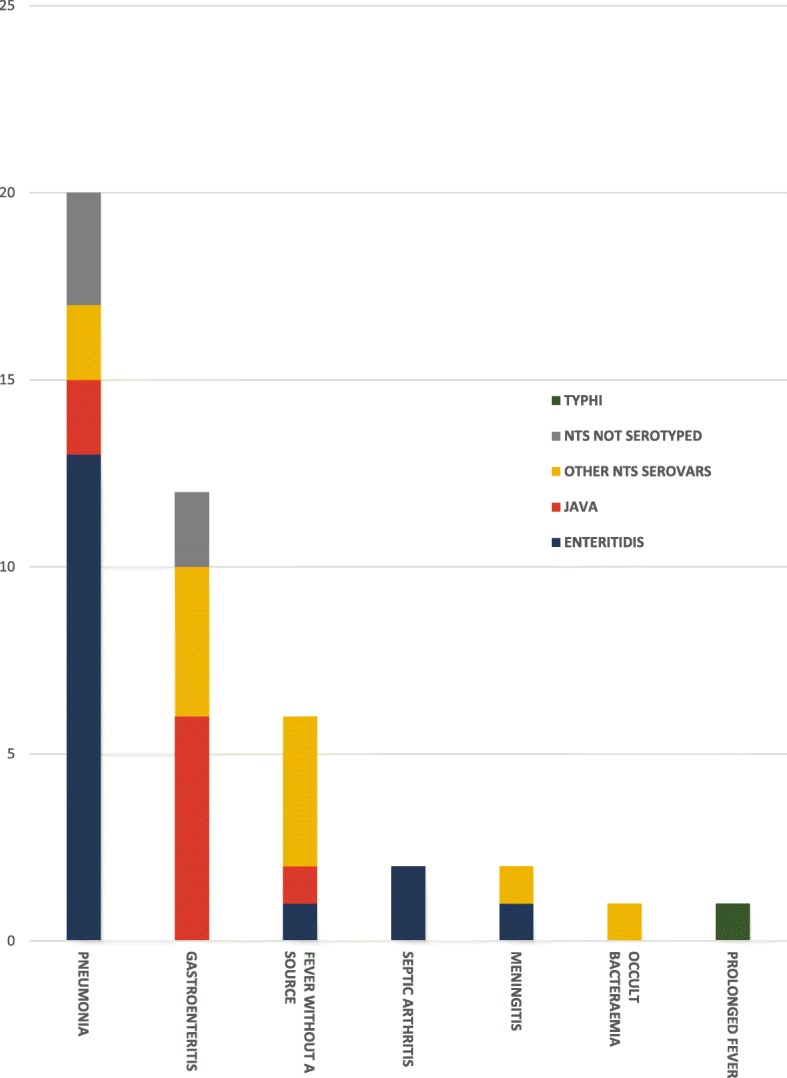
Distribution of invasive *Salmonella* infections among children in Bintulu according to clinical manifestation and serovar. The bar chart depicts the distribution of the 44 invasive *Salmonella* cases identified in the study, according to the clinical manifestation at presentation, with the number of cases attributed to the various serovars shown

Pneumonia was the most common clinical manifestation (Fig. 3), present in 20 (47%) of the 43 children with iNTS. Among those with pneumonia, *Salmonella* Enteritidis was identified in 13 (76%) of 17 children whose NTS isolates were serotyped, significantly more frequently than in children with other clinical manifestations (13/17 [76%] vs 4/21 [19%], *P* = 0.001, OR 13.9, 95%CI 2.9–66.7). Children with *Salmonella* Enteritidis infection presented predominantly with fever and respiratory symptoms and signs; gastro-intestinal symptoms and signs were uncommon (Table 3). Among 17 (85%) children with pneumonia whose chest radiograph findings were recorded, 12 (71%) had unilateral or bilateral peri-hilar interstitial haziness, 2 (12%) had unilateral or bilateral alveolar opacities while the remaining 3 (18%) had clear lung fields.

**Table 3:** Presentation of children with invasive non-typhoidal *Salmonella* infections by serovar in the 38 children whose isolates were serotyped

Characteristic	All serovars	*Salmonella* Enteritidis	*Salmonella* Java	Other NTS serovars	*P*
No. of patients	38	17	9	12	–
Presenting symptoms					
Time between onset and admission, days, median (IQR)	2 (2–5)	3 (2–6)	3 (2–8)	2 (1–4)	0.1
Fever	34 (89)	16 (94)	7 (78)	11 (92)	0.4
Poor appetite	26 (68)	12 (71)	5 (56)	9 (75)	0.7
Lethargy or irritability	23 (61)	9 (53)	5 (56)	9 (75)	0.5
Cough	20 (53)	13 (77)	3 (33)	4 (33)	0.03
URTI symptoms	17 (45)	10 (59)	3 (33)	4 (33)	0.4
Diarrhea	15 (40)	4 (24)	6 (67)	5 (42)	0.1
Vomiting	5 (13)	1 (6)	2 (22)	2 (17)	0.5
Presenting signs					
Signs of respiratory distress	26 (68)	15 (88)	4 (44)	7 (58)	0.05
Tachycardia for age	24 (63)	13 (77)	6 (67)	5 (42)	0.2
Tachypnoea for age	20 (53)	13 (77)	5 (56)	2 (17)	0.007
Signs of dehydration	15 (40)	4 (24)	5 (56)	6 (50)	0.2
Abnormal lung findings^a^	13 (34)	9 (53)	1 (11)	3 (25)	0.1
Hepatomegaly	9 (24)	7 (41)	0 (0)	2 (17)	0.08
Pallor	7 (18)	4 (24)	1 (11)	2 (17)	0.9
Poor perfusion	6 (16)	3 (18)	2 (22)	1 (8)	0.7
Splenomegaly	2 (5)	2 (12)	0 (0)	0 (0)	0.5
Investigations^b^					
Hemoglobin, g/dL, median (IQR)	11.4 (10.1–12.2)	11.5 (10.4–12.2)	11.4 (10.2–14.0)	10.5 (9.4–12.7)	0.5
WBC, X 10^9^ cells/L, median (IQR)	13.3 (9.7–18.3)	13.3 (9.5–17.6)	17.2 (10.3–20.3)	13.2 (8.5–17.6)	0.5
Neut, X 10^9^ cells/L, median (IQR)	5.7 (4.2–13.5)	5.7 (3.6–9.1)	6.4 (4.8–15.2)	6.2 (4.2–14.3)	0.7
Lymph, X 10^9^ cells/L, median (IQR)	5.4 (3.8–7.5)	5.8 (4.7–9.3)	5.8 (3.8–10.7)	4.4 (2.7–6.9)	0.3
Platelet, X 10^9^/L, median (IQR)	343 (251–463)	362 (185–459)	462 (313–492)	347 (238–487)	0.4
ESR, mm/hr., median (IQR)	32 (10–48)	32 (10–41)	50 (24–75)	23 (6–61)	0.6
Sodium, mmol/L, median (IQR)	136 (133–138)	137 (135–139)	134 (129–137)	135 (133–137)	0.1
Urea, mmol/L, median (IQR)	3.1 (2.3–4.3)	2.7 (2.3–3.6)	3.5 (2.8–7.3)	3.4 (2.3–5.1)	0.4
Albumin, g/L, median (IQR)	38 (35–44)	36 (34–44)	45 (43–46)	41 (34–46)	0.3
AST, U/L, median (IQR)	51 (40–108)	49 (40–244)	98 (88–108)	48 (34–58)	0.4
ALT, U/L median (IQR)	22 (16–90)	22 (16–82)	97 (85–109)	19 (15–94)	0.4

Data are No. (%) unless otherwise indicated

^a^ All children with abnormal lung examination had bilateral findings: 6 had both rhonchi and crepitation, 6 only rhonchi and 1 only crepitation

^b^Only investigations done on the day of admission were included: 37 children had Hemoglobin, WBC, Platelet (Enteritidis, n = 17; Java, n = 8; Other serovars, n = 12); 27 children had differential counts (Enteritidis, n = 12; Java, n = 7; Other serovars, n = 8); 13 children had ESR (Enteritidis, n = 7; Java, n = 2; Other serovars, n = 4); 37 children had Sodium, Urea (Enteritidis, n = 17; Java, n = 9; Other serovars, n = 11); 15 children had Albumin, AST, ALT (Enteritidis, n = 9; Java, n = 2; Other serovars, n = 4)

Abbreviations: *IQR* interquartile range, *URTI* upper respiratory tract infection, *WBC* white blood cell count, *Neut* neutrophil count, *Lymph* lymphocyte count, *ESR* erythrocyte sedimentary rate, *AST* aspartate aminotransferase, *ALT* alanine aminotransferase

Twelve (28%) children with iNTS presented with gastroenteritis (Fig. 3). Of the 10 whose isolates were serotyped, *Salmonella* Java and *Salmonella* Corvallis were identified in 6 (60%) and 3 (30%) children respectively. *Salmonella* Java was identified more frequently in children with gastroenteritis than in those with other diagnoses (6/10 [60%] vs 3/28 [11%], *P* = 0.005, OR 12.5, 95%CI 2.2–71.4). Fever and watery diarrhea were the most common presenting features, noted in 11 (92%) and 10 (83%) of the 12 children respectively. Only 2 (17%) had blood in stools. Stool cultures were performed in only 4 (33%) of these children; all were negative.

Two (5%) children had NTS meningitis, one caused by *Salmonella* Enteritidis and the other by *Salmonella* Poona. Both infants were < 2 months of age. Meningitis was significantly more common in this age group than in older infants and children (2/7 [29%] vs 0/36 [0%], *P* = 0.02). Both infants presented with an abrupt onset of fever and lethargy and rapidly developed septicaemic shock on the same day. Lumbar puncture was only performed after 3 and 7 days of admission respectively, due to the unstable cardio-respiratory condition. Both had abnormal CSF examination: 558 white blood cells/mm^3^ (80% polymorphs) and 41 white blood cells/mm^3^ (5% polymorphs) respectively. Twenty-seven (66%) of the remaining 41 children with NTS infections (who presented primarily with respiratory or gastro-intestinal manifestations) had lumbar puncture; all except 1 (who had a traumatic procedure) had normal CSF examination.

Two (5%) children presented with septic arthritis, both due to *Salmonella* Enteritidis. Both had unilateral hip joint involvement: one was a 5-month old infant without any risk factor while the other was an 11-year old with relapsed acute leukaemia on palliative chemotherapy.

A 16-month old girl with West syndrome and swallowing difficulty requiring nasogastric tube feeding had occult bacteraemia with *Salmonella* Ohio. She presented to hospital with increased oropharyngeal secretions but had no fever or other localizing symptoms or signs.

### Antimicrobial susceptibility

Among NTS isolates, in vitro resistance to tetracycline, ampicillin, chloramphenicol and cotrimoxazole was noted in 61% (25/41), 21% (9/43), 21% (9/43) and 5% (2/43) respectively. All isolates were susceptible to ciprofloxacin and ceftriaxone. Sixteen (94%) of the 17 *Salmonella* Enteritidis isolates were susceptible to ampicillin, whereas only 2 (22%) of the 9 *Salmonella* Java isolates were susceptible to this antimicrobial agent (Table 4). Multi-drug resistance was observed more frequently in *Salmonella* Java isolates than in *Salmonella* Enteritidis isolates (7/9 [78%] vs 1/17 [6%], *P* < 0.001, OR 56, 95%CI 4.3–724).

**Table 4:** Antimicrobial resistance of non-typhoidal *Salmonella* isolates by serovar

Antimicrobial	All serovars *n/N* (%)	*Salmonella* Enteritidis^a^ *n/N* (%)	*Salmonella* Java^a^ *n/N* (%)	Other NTS serovars^a^ *n/N* (%)
Tetracycline^b^	22/37 (59)	5/16 (31)	7/9 (78)	10/12 (83)
Ampicillin	9/38 (24)	1/17 (6)	7/9 (78)	1/12 (8)
Chloramphenicol	9/38 (24)	1/17 (6)	7/9 (78)	1/12 (8)
Cotrimoxazole	2/38 (5)	1/17 (6)	0/9 (0)	1/12 (8)
Ciprofloxacin	0/38 (0)	0/17 (0)	0/9 (0)	0/12 (0)
Ceftriaxone	0/38 (0)	0/17 (0)	0/9 (0)	0/12 (0)

^a^Multi-drug resistance was found in 9 isolates (*Salmonella* Enteritidis, n = 1; *Salmonella* Java, n = 7; *Salmonella* Ohio, n = 1)

^b^Tetracycline susceptibility was not recorded in 1 *Salmonella* Enteritidis isolate

The sole *Salmonella* Typhi isolate was susceptible to all antimicrobials tested, including tetracycline, ampicillin, chloramphenicol, cotrimoxazole, ciprofloxacin and ceftriaxone.

### Case management and outcome

Eleven (26%) children with iNTS required intensive care: both (100%) children with meningitis, 8 (40%) of 20 with pneumonia and 1 (8%) of 12 with gastroenteritis. Reasons for intensive care included impending respiratory failure (*n* = 7) and septicaemic shock (*n* = 4). *Salmonella* Enteritidis was identified in 8 (73%) of these children. Forty-seven percent of all children with invasive *Salmonella* Enteritidis infection required intensive care.

Invasive NTS infections resulted in death or severe sequelae in 4 (9%) children. There were 2 fatalities: one was a 4-week old infant with meningitis who had severe neurological deficits, remained ventilator-dependent after 15 days in intensive care and died when respiratory support was withdrawn; the other was a 4-month old who had pneumonia complicated by hemophagocytic lymphohistiocytosis. The 2 children who survived with severe sequelae included a 6-week old infant with meningitis (microcephaly, global developmental delay, infantile spasms and cortical blindness present at discharge from hospital at age 3.5 months and persisting on follow up at age 2 years) and a 3-month old with pneumonia who had limb amputations associated with purpura fulminans. Of these 4 cases, 3 (75%) had *Salmonella* Enteritidis and 1 (the infant with meningitis surviving with severe sequelae) had *Salmonella* Poona infection.

All children received parenteral antibiotics. Ampicillin was the most common empiric antibiotic used, initiated in 24 (55%) children. Other initial antibiotics, given either empirically or upon detection of Gram negative bacteraemia, included ceftriaxone (*n* = 6), cefuroxime (*n* = 4), ceftazidime (n = 4), ampicillin-sulbactam (n = 4), cefotaxime (*n* = 1) and benzyl penicillin (n = 1). Of those given ampicillin empirically, 3 were found to have non-susceptible isolates, requiring a switch to ceftriaxone. Thirty-five (80%) children ultimately received ceftriaxone, mainly to facilitate ambulatory care. The median duration of parenteral treatment was 14 (IQR 6–18) days. In 27 children, treatment was further extended with an oral antibiotic, mainly amoxicillin or cotrimoxazole. The median total duration of antibiotics was 21 (IQR 12–43) days.

Among survivors, fever resolved rapidly (within a median of 1 [IQR 1–2] day) following initiation of an appropriate antibiotic. Only 1 recurrent infection was recorded. This was in a 6 month old with an invasive *Salmonella* Enteritidis infection (presenting with pneumonia), treated with a total of 13 days of antibiotics (5 days of parenteral, 8 days oral). Ten days after completion of antibiotics, fever recurred. *Salmonella* Enteritidis was again cultured from blood.

## Discussion

To our knowledge, this study is the first detailed report of invasive *Salmonella* infections among children in Sarawak, Malaysian Borneo, and shows a striking and underappreciated burden of iNTS disease, and conversely, a rarity of typhoidal *Salmonella* infections in this population. It also highlights important and previously undescribed clinical and bacterial characteristics of iNTS infections in this region.

The incidence of childhood iNTS infections found in this study were among the highest recorded outside of sub-Saharan Africa. The average annual incidence of 32.4 per 100,000 children aged < 5 years and 135.1 per 100,000 aged < 1 year in Bintulu were markedly higher than the iNTS incidences recorded at sites in all 5 Asian countries included in the multicentre community-based prospective study, namely Pakistan (7.2 per 100,000 aged 2–15 years), India (1.8 per 100,000), Indonesia (1.0 per 100,000), China (0.0 per 100,000 aged 5–60 years) and Viet Nam (0.0 per 100,000 aged 5–18 years) [7]. In fact, the average annual iNTS incidence in Bintulu was only slightly lower than the incidences in some areas of sub-Saharan Africa, including Mozambique (217.7 per 100,000 children aged < 1 year) and Kenya (36.6 per 100,000 children aged < 5 years) [16, 17]. The higher incidence found in Bintulu in comparison to the Asian sites may be due to the fact that children under 2 years, who typically have a higher risk of iNTS infection, were included in only 2 of the 5 sites of the multicentre study [7]. However, other unknown epidemiological factors may also be contributing to the higher incidence in Bintulu. Importantly, the iNTS incidence reported in the present study likely remains an under-estimate of the true population incidence as this hospital based study has only identified severe cases that resulted in hospitalization. Our findings are however consistent with those of a study estimating the global burden of iNTS which predicted an incidence of 5–92 cases per 100,000 population in Southeast Asia, and lends support to the conclusions that iNTS is a major but under-recognized cause of bacteraemic illness globally [18].

Intriguingly, the high incidence of childhood iNTS disease recorded in this study occurred in a population not burdened by malnutrition, malaria or HIV infection, unlike the iNTS infections described in most other regions [5, 19, 20]. For example, 31% of children with iNTS in Mozambique were severely malnourished while 43% had malaria [16]. In Bintulu, only 7% of children with iNTS were severely malnourished, and none were diagnosed to have malaria or HIV infection. Although 30% did have an underlying medical condition, the significance of this finding is unknown as the medical conditions, with the exception of acute leukaemia, were not commonly known risk factors of NTS infection. None of the children were screened for the presence of underlying primary immunodeficiency conditions, and this remains a limitation of the study.

*Salmonella* Enteritidis and *Salmonella* Java were the most common NTS serovars identified in Bintulu, causing 45 and 24% of the iNTS infections respectively. In Africa, *Salmonella* Enteritidis has been implicated in around 20% of iNTS cases, including 36% of childhood cases in Mali [5, 21]. Similarly, in Asia, *Salmonella* Enteritidis was identified in > 40% of iNTS cases in Vietnam and Peninsular Malaysia, confirming the global distribution and importance of this serovar in causing invasive disease [20, 22]. This is in contrast to the distribution of *Salmonella* Typhimurium, the other major serovar implicated in iNTS infections, responsible for almost 70% of cases in Africa [5]. No *Salmonella* Typhimurium iNTS infections were noted in Bintulu. Similarly, very few *Salmonella* Typhimurium iNTS cases were found in previous studies in Peninsular Malaysia [22, 23]. The reasons for this geographical variation in serovar distribution are not known, and further epidemiological studies are required.

Invasive *Salmonella* Enteritidis infections in Bintulu manifested with a wide spectrum of clinical presentations, foremost of which was pneumonia, and almost half resulted in severe disease. Pneumonia was the primary presentation in 76% of children with *Salmonella* Enteritidis bacteraemia, with the remainder presenting with septic arthritis, meningitis or fever without a source. Studies of iNTS disease in children in Africa have also reported respiratory presentations in large proportions of cases [24, 25]. In Ghana, NTS was shown to be the predominant organism isolated in children with clinical pneumonia [26]. In Thailand, clinical pneumonia was diagnosed in 25% of children with iNTS disease [27]. Interestingly, 82% of these children had abnormal, predominantly interstitial, infiltrates on the chest radiograph; a finding similarly noted in the present study. Whether these pulmonary manifestation are due solely to NTS or is instead due to infection with other more typical but undiagnosed viral or bacterial respiratory pathogens, either singly or as a part of a co-infection with the NTS, remains unknown. In the present study, almost 50% of children with *Salmonella* Enteritidis bacteraemia had severe disease necessitating intensive care. Death or long-term morbidity occurred in 18%, highlighting the considerable burden of these infections. In addition, long-term pulmonary sequelae, not evaluated in this study, could also have occurred.

In contrast to the *Salmonella* Enteritidis infections, invasive *Salmonella* Java infections manifested primarily with gastro-intestinal disease. *Salmonella* Java, described as the d-tartrate fermenting biovar of *S. enterica* subspecies enterica serovar Paratyphi B, is thought to be less virulent compared to the non d-tartrate fermenting variant (classical *Salmonella* Paratyphi B), with infections typically localized to the gastro-intestinal tract [28]. Large outbreaks of these gastro-intestinal tract infections have been reported, resulting mainly from contaminated food products [29]. Although invasive infections have been documented, *Salmonella* Java has not previously been reported to be a major cause of invasive disease [30, 31]. It is unclear whether the frequent bacteraemia found in the present study is due to increased invasiveness or is in fact a reflection of a large, unrecognized burden of gastro-intestinal *Salmonella* Java infections as the epidemiology of acute gastro-enteral infections in this population has not been studied.

NTS meningitis occurred more frequently in very young infants and resulted in severe morbidity and mortality. Both infants in this study were < 2 months of age, with one having a fatal outcome and the other suffering severe neurological sequelae. Previous studies have documented similar findings. Among non-HIV infected individuals in South Africa, NTS meningitis occurred only in those aged < 5 years [32]. Of these, 41% were < 2 months of age. Children with NTS meningitis in Kuala Lumpur had a mortality of 18%, with a further 36% having severe long-term sequelae [33]. In the present study, 67% of all children had CSF examination, despite the majority presenting with respiratory or gastro-intestinal manifestations without overt symptoms or signs of meningitis. CSF findings were normal in all except the 2 infants described above. These findings support a recently published recommendation that CSF be examined in all infants aged < 3 months diagnosed with NTS bacteraemia, but only when clinically indicated in older infants [34].

In regions with a high prevalence of iNTS infection, *Salmonella* has been reported to be the leading cause of septic arthritis in children [35]. This is in stark contrast to data from regions with low iNTS prevalence, where *Staphylococcus* and *Streptococcus* (and some Gram negative bacilli in the neonatal period) are the predominant causes of childhood septic arthritis. In the present study, 2 cases of septic arthritis were identified, possibly indicating a similar importance of NTS in the etiology of septic arthritis in Bintulu Division.

Apart from *Salmonella* Java isolates, nearly all other NTS isolates in the study were susceptible to most antimicrobials, including ampicillin. Worldwide, an increasing prevalence of antimicrobial resistance in NTS has been reported [1]. In Southeast Asia, resistance to ampicillin was documented in 31 and 68% of NTS isolates in Penang (Malaysia) and Bangkok (Thailand), respectively [23, 27]. Multi-drug resistance was documented in 48% of isolates in sub-Saharan Africa [6]. In Bintulu, multi-drug resistance was found mainly in *Salmonella* Java, with 78% of isolates noted to be multi-drug resistant. These isolates, only detected since 2014, had identical resistance spectrums consisting of ampicillin, chloramphenicol and tetracycline. Although the susceptibility to streptomycin and sulfonamides are not known (resistance to these antimicrobials are not routinely screened for in Bintulu), it is possible that these *Salmonella* Java isolates contain Salmonella genomic island 1 (SGI1), which typically harbor genes that confer resistance to these antimicrobials [36]. Multi-drug resistant *Salmonella* Java containing SGI1, believed to be clonal in origin, has been reported globally [37, 38]. The high prevalence of multi-drug resistance among the *Salmonella* Java isolates in Bintulu suggests either the dominance of the drug-resistant strain in this region, an increased predisposition to severe human infections or the presence of a common epidemiological source of infection in the patients. Studies to determine the complete drug-resistance profile, molecular typing, and source of the *Salmonella* Java infections are urgently needed. As *Salmonella* Java was less likely to be associated with respiratory presentations than *Salmonella* Enteritidis, the continued use of amino-penicillins in the empiric treatment of pneumonia among children in Bintulu, as is recommended in most guidelines, remains appropriate [39].

## Conclusions

Bintulu Division in Sarawak, Malaysian Borneo has an important yet under-appreciated burden of childhood iNTS infections. Severe disease occurred in over 25%, with long-term morbidity and mortality in nearly 10% of cases, despite most children having no commonly known risk for infection. *Salmonella* Enteritidis was the commonest NTS serovar identified, with affected children presenting primarily with pneumonia. Apart from *Salmonella* Java, nearly all isolates were susceptible to most antimicrobials, including ampicillin.

## Abbreviations

CI: Confidence interval
CSF: Cerebrospinal fluid
HIV: Human immunodeficiency virus
iNTS: Invasive non-typhoidal *Salmonella*
IQR: Interquartile range
NTS: Non-typhoidal *Salmonella*
OR: Odds ratio
SGI1: Salmonella genomic island 1
WHO: World Health Organization

## References

[1] Crump JA, Sjolund-Karlsson M, Gordon MA, Parry CM (2015). Epidemiology, clinical presentation, laboratory diagnosis, antimicrobial resistance, and antimicrobial Management of Invasive Salmonella Infections. Clin Microbiol Rev.

[2] Crump JA, Heyderman RSA (2015). Perspective on invasive Salmonella disease in Africa. Clinical infectious diseases : an official publication of the Infectious Diseases Society of America.

[3] Majowicz SE, Musto J, Scallan E, Angulo FJ, Kirk M, O'Brien SJ (2010). The global burden of nontyphoidal Salmonella gastroenteritis. Clinical infectious diseases : an official publication of the Infectious Diseases Society of America.

[4] Feasey NA, Dougan G, Kingsley RA, Heyderman RS, Gordon MA (2012). Invasive non-typhoidal salmonella disease: an emerging and neglected tropical disease in Africa. Lancet.

[5] Uche IV, MacLennan CA, Saul A (2017). A systematic review of the incidence, risk factors and case fatality rates of invasive Nontyphoidal Salmonella (iNTS) disease in Africa (1966 to 2014). PLoS Negl Trop Dis.

[6] Marks F, von Kalckreuth V, Aaby P, Adu-Sarkodie Y, El Tayeb MA, Ali M (2017). Incidence of invasive salmonella disease in sub-Saharan Africa: a multicentre population-based surveillance study. Lancet Glob Health.

[7] Khan MI, Ochiai RL, von Seidlein L, Dong B, Bhattacharya SK, Agtini MD (2010). Non-typhoidal Salmonella rates in febrile children at sites in five Asian countries. Tropical medicine & international health : TM & IH.

[8] Ochiai RL, Acosta CJ, Danovaro-Holliday MC, Baiqing D, Bhattacharya SK, Agtini MD (2008). A study of typhoid fever in five Asian countries: disease burden and implications for controls. Bull World Health Organ.

[9] Nor Azizah A, Fadzilah MN, Mariam M, Anis Siham ZA, Ariza A, Noor Shafina MN (2016). Community-acquired bacteremia in Paediatrics: epidemiology, aetiology and patterns of antimicrobial resistance in a tertiary care Centre, Malaysia. Med J Malaysia.

[10] Ministry of Health Malaysia. Health facts 2017 (reference data for 2016). 2017. [https://myhdw.moh.gov.my/public/documents/20186/150084/HEALTH+FACTS+2017/98041185-ce34-4877-9ea1-4d5341e43187?version=1.1&download=true] Accessed 31 March 2018.

[11] WHO. The WHO Child Growth Standards. In: Programmes and projects. 2018. [http://www.who.int/childgrowth/en/] Accessed 31 March 2018.

[12] WHO. Growth reference data for 5–19 years. In: Programmes and projects. 2018. [http://www.who.int/growthref/en/] Accessed 31 March 2018.

[13] WHO. Pocket Book of Hospital care for children: Guideline for the management of common illnesses with limited resources. WHO press; 2005.

[14] El-Tayeb MA, Ibrahim ASS, Al-Salamah AA, Almaary KS, Elbadawi YB (2017). Prevalence, serotyping and antimicrobials resistance mechanism of Salmonella enterica isolated from clinical and environmental samples in Saudi Arabia. Brazilian journal of microbiology : [publication of the Brazilian Society for Microbiology].

[15] Malorny B, Bunge C, Helmuth R (2003). Discrimination of d-tartrate-fermenting and-nonfermenting Salmonella enterica subsp. enterica isolates by genotypic and phenotypic methods. J Clin Microbiol.

[16] Mandomando I, Bassat Q, Sigauque B, Massora S, Quinto L, Acacio S (2015). Invasive Salmonella infections among children from rural Mozambique, 2001-2014. Clinical infectious diseases : an official publication of the Infectious Diseases Society of America.

[17] Muthumbi E, Morpeth SC, Ooko M, Mwanzu A, Mwarumba S, Mturi N (2015). Invasive Salmonellosis in Kilifi, Kenya. Clinical infectious diseases : an official publication of the Infectious Diseases Society of America.

[18] Ao TT, Feasey NA, Gordon MA, Keddy KH, Angulo FJ, Crump JA. Global burden of invasive nontyphoidal Salmonella disease, 2010(1). Emerg Infect Dis. 2015;21(6). 10.3201/eid2106.140999 PMC4451910 25860298

[19] Park SE, Pak GD, Aaby P, Adu-Sarkodie Y, Ali M, Aseffa A (2016). The relationship between invasive Nontyphoidal Salmonella disease, other bacterial bloodstream infections, and malaria in sub-Saharan Africa. Clinical infectious diseases : an official publication of the Infectious Diseases Society of America.

[20] Phu Huong Lan N, Le Thi Phuong T, Nguyen Huu H, Thuy L, Mather AE, Park SE (2016). Invasive non-typhoidal Salmonella infections in Asia: clinical observations, disease outcome and dominant Serovars from an infectious disease Hospital in Vietnam. PLoS Negl Trop Dis.

[21] Tapia MD, Tennant SM, Bornstein K, Onwuchekwa U, Tamboura B, Maiga A (2015). Invasive Nontyphoidal Salmonella infections among children in Mali, 2002-2014: microbiological and epidemiologic features guide vaccine development. Clinical infectious diseases : an official publication of the Infectious Diseases Society of America.

[22] Lee WS, Puthucheary SD, Parasakthi N, Choo KE (2003). Antimicrobial susceptibility and distribution of non-typhoidal Salmonella serovars isolated in Malaysian children. J Trop Pediatr.

[23] Dhanoa A, Fatt QK (2009). Non-typhoidal Salmonella bacteraemia: epidemiology, clinical characteristics and its' association with severe immunosuppression. Ann Clin Microbiol Antimicrob.

[24] MacLennan CA, Msefula CL, Gondwe EN, Gilchrist JJ, Pensulo P, Mandala WL (2017). Presentation of life-threatening invasive nontyphoidal Salmonella disease in Malawian children: a prospective observational study. PLoS Negl Trop Dis.

[25] Brent AJ, Oundo JO, Mwangi I, Ochola L, Lowe B, Berkley JA (2006). Salmonella bacteremia in Kenyan children. Pediatr Infect Dis J.

[26] Schwarz NG, Sarpong N, Hunger F, Marks F, Acquah SE, Agyekum A (2010). Systemic bacteraemia in children presenting with clinical pneumonia and the impact of non-typhoid salmonella (NTS). BMC Infect Dis.

[27] Punpanich W, Netsawang S, Thippated C (2012). Invasive salmonellosis in urban Thai children: a ten-year review. Pediatr Infect Dis J.

[28] Chart H (2003). The pathogenicity of strains of Salmonella paratyphi B and Salmonella java. J Appl Microbiol.

[29] Gobin M, Launders N, Lane C, Kafatos G, Adak B (2011). National outbreak of Salmonella Java phage type 3b variant 9 infection using parallel case-control and case-case study designs, United Kingdom, July to October 2010. Euro surveillance : bulletin Europeen sur les maladies transmissibles = European communicable disease bulletin.

[30] Koch K, Kristensen B, Holt HM, Ethelberg S, Molbak K, Schonheyder HC (2011). International travel and the risk of hospitalization with non-typhoidal Salmonella bacteremia. A Danish population-based cohort study, 1999-2008. BMC Infect Dis.

[31] Laupland KB, Schonheyder HC, Kennedy KJ, Lyytikainen O, Valiquette L, Galbraith J (2010). Salmonella enterica bacteraemia: a multi-national population-based cohort study. BMC Infect Dis.

[32] Keddy Karen H., Sooka Arvinda, Musekiwa Alfred, Smith Anthony M., Ismail Husna, Tau Nomsa P., Crowther-Gibson Penny, Angulo Frederick J., Klugman Keith P. (2015). Clinical and Microbiological Features ofSalmonellaMeningitis in a South African Population, 2003–2013. Clinical Infectious Diseases.

[33] Lee WS, Puthucheary SD, Omar A (1999). Salmonella meningitis and its complications in infants. J Paediatr Child Health.

[34] Wen SC, Best E, Nourse C (2017). Non-typhoidal Salmonella infections in children: review of literature and recommendations for management. J Paediatr Child Health.

[35] Lavy CB (2007). Septic arthritis in Western and sub-Saharan African children-a review. Int Orthop.

[36] Levings RS, Lightfoot D, Partridge SR, Hall RM, Djordjevic SP (2005). The genomic island SGI1, containing the multiple antibiotic resistance region of Salmonella enterica serovar typhimurium DT104 or variants of it, is widely distributed in other S. enterica serovars. J Bacteriol.

[37] Djordjevic SP, Cain AK, Evershed NJ, Falconer L, Levings RS, Lightfoot D (2009). Emergence and evolution of multiply antibiotic-resistant Salmonella enterica serovar Paratyphi B D-tartrate-utilizing strains containing SGI1. Antimicrob Agents Chemother.

[38] Mulvey MR, Boyd D, Cloeckaert A, Ahmed R, Ng LK (2004). Emergence of multidrug-resistant Salmonella Paratyphi B dT+, Canada. Emerg Infect Dis.

[39] Ministry of Health Malaysia (2008). National Antibiotic Guideline. Ministry of Health Malaysia.

